# Dietary polyphenols enhance optogenetic recall of fear memory in hippocampal dentate gyrus granule neuron subpopulations

**DOI:** 10.1038/s42003-018-0043-5

**Published:** 2018-05-03

**Authors:** Chad Smith, Tal Frolinger, Justin Brathwaite, Steven Sims, Giulio M. Pasinetti

**Affiliations:** 1 Department of Neurology, Icahn School of Medicine at Mount Sinai, New York, 10029 NY USA; 2 JJ Peters VA Medical Center, Bronx, 10468 VA USA

## Abstract

Grape-derived polyphenols have been investigated for their role in promoting memory in model systems of stress, but little is known about select subpopulations of neurons that are influenced by polyphenols to improve memory performance. Granule neurons in the hippocampal dentate gyrus are vulnerable to stressors that impair contextual memory function and can be influenced by dietary polyphenols. We utilized a c-fos-tTA/TRE-ChR2 optogenetics model in which neurons activated during fear learning are labeled with ChR2-mCherry and can be optically reactivated in a different context to recapitulate the behavioral output of a related memory. Treatment with dietary polyphenols increased fear memory recall and ChR2-mCherry expression in dentate gyrus neurons, suggesting that dietary polyphenols promote recruitment of neurons to a fear memory engram. We show that dietary polyphenols promote memory function and offer a general method to map cellular subpopulations influenced by dietary polyphenols, in part through the mechanism of c-Fos expression enhancement.

## Abstract (web summary)

Chad Smith et al. show that dietary polyphenols, compounds found in grapes, enable mice to remember fearful events more effectively and map this function to the hippocampal dentate gyrus neurons. This study offers a way to identify the cellular subpopulations regulated by dietary polyphenols.

## Introduction

Polyphenolic compounds from grapes and other sources are receiving considerable attention with regards to promoting cognitive function and physiological resilience against cognitive deficits from stress and neurological disorders^1–5^. A bioactive dietary polyphenol preparation (BDPP) comprised of grape seed polyphenol extract (GSPE), concord grape juice (CGJ), and resveratrol (RSV) has been found to enhance performance in hippocampus-dependent memory tasks under conditions of stress and neurodegeneration^5–8^. Evidence suggests that dietary polyphenols confer benefits though modulations in synaptic plasticity, inflammatory pathways, and promotion of neurogenesis, among other mechanisms; however, the precise mechanisms through which polyphenols confer benefits in cognitive and memory function are not clearly understood^9–12^.

Among the regions influenced by treatment with dietary polyphenols is the hippocampal formation, which is involved in several functions of learning and cognition, including episodic memory^6,13^. The dorsal hippocampus plays an essential role in the encoding and retrieval of contextual and spatial memory, and the ventral hippocampus is crucial in the regulation of anxiety responses and exploratory drive^14–16^. Granule neurons in the hippocampal dentate gyrus (DG) have been assigned an essential role in discriminating between similar environmental contexts^17^. Studies of immediate early gene (IEG) expression show that sparse subpopulations of DG granule neurons (~2%) are activated in a distinct environmental context^18^; and upon re-exposure to the same context, the same subset of DG granule neurons are re-activated. Therefore, different environments activate different subsets of DG granule neurons. Optically reactivating sparse subpopulations of dorsal DG neurons activated by behavioral trials in a distinct context recapitulates behavior learned in that context, even with the absence of the stimuli that previously activated these neurons^19–22^. These observations point toward the DG as an ideal target for the investigation of dietary polyphenols in the promotion of contextual memory function.

Previous optogenetics studies were based on the expression of the IEGs *c-fos* and *Arc*, transcription factors that are rapidly and transiently expressed following synaptic stimulation through behavioral tasks^23–26^. The expression of IEGs is commonly used as a marker of neural activity and as a means to identify neuronal subpopulations that form a component of a memory engram^24^.

We previously found that dietary polyphenols promote hippocampus-dependent memory function through activation of the CaMKII-CREB and mTOR signaling pathways and that they were able to attenuate memory impairments under stressful conditions and models of neurodegeneration^5–7,27^. The modulation of CaMKII-CREB signaling by specific bioavailable phenolic metabolites suggests that upregulation of IEGs, including c-Fos, may be among the mechanisms through which dietary polyphenols promote memory function^7,28^. Based in part on this, we utilized an inducible c-fos-tTA/TRE-ChR2 optogenetics model to identify subpopulations of DG granule neurons that are influenced by polyphenol treatment and recapitulate learned behavior in the same animal^19,20^.

Here, we show that BDPP treatment in c-fos-tTA/TRE-ChR2-mCherry mice results in increased *c-fos-*promoter-induced expression of ChR2-mCherry and endogenous c-Fos, as well as increased recapitulation of fear memory upon optical stimulation in a distinct context. These findings suggest that the promotion of memory function by dietary polyphenols may, in part, be causally associated with increased c-Fos expression and recruitment of hippocampal DG granule neurons to a memory engram.

## Results

**Fig. 1:**
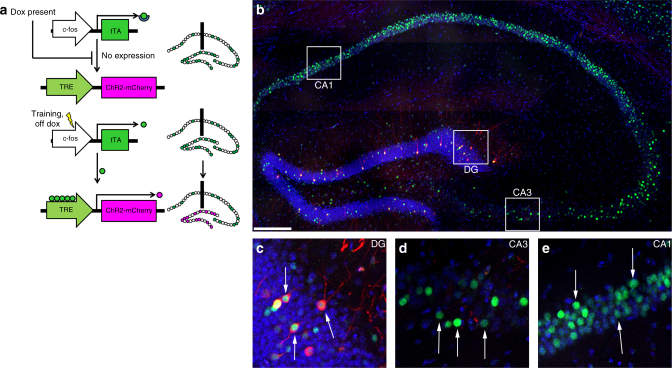
Expression of ChR2-mCherry is restricted to the DG. **a** Expression of ChR2-mCherry in presence or absence of Dox. In the presence of Dox, tTA expressed through the *c-fos* promoter is unable to bind to the TRE promoter, suppressing expression of ChR2-mCherry. Withdrawal of Dox opens a window in which training labels a subpopulation of activated neurons with ChR2-mCherry. **b** Hippocampal section of a representative mouse after Dox withdrawal and FC training. Although a subpopulation of neurons in the entire hippocampus express c-Fos (DG, CA3, CA1; **c**–**e**), expression of ChR2-mCherry is restricted to the DG (**c**). Scale bar: 200 µm. Arrows: representative neurons

To label and optically reactivate a subpopulation of hippocampal DG granule neurons, which were activated during memory encoding, we stereotaxically injected the DG of c-fos-tTA transgenic mice with AAV_9_-TRE-ChR2-mCherry and implanted the mice with optical fibers targeting the injection site. This approach restricts the expression of ChR2-mCherry to neurons in which the IEG *c-fos* is expressed in the absence of Dox^24,29^. In this system, treatment with Dox inhibits *c-fos-*promoter-driven tTA from driving the expression of ChR2-mCherry by preventing it from binding to the tetracycline-response element (Fig. 1a). When Dox is withdrawn, c-Fos-expressing activated neurons are selectively labeled with ChR2-mCherry, which can be reactivated via optical stimulation during subsequent trials. Fluorescent microscopy confirmed that our injection limited the expression of ChR2-mCherry to DG granule neurons in the hippocampus (Fig. 1b, c, d, e).

### Timeline of activity-dependent labeling

**Fig. 2:**
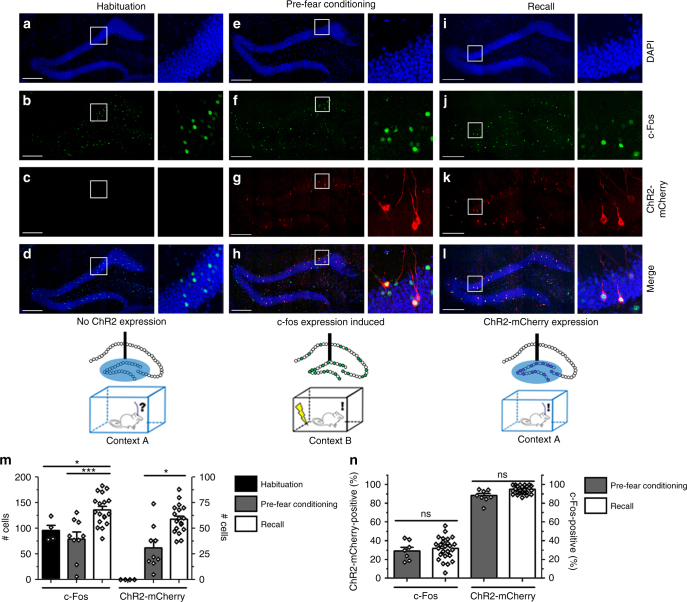
Expression of ChR2-mCherry and c-Fos in c-fos-tTA/TRE-ChR2 optogenetics model. **a**–**l** Timeline of ChR2-mCherry expression. Mice are trained through CFC and sacrificed at the following points: after habituation (**a**–**d**), after 2 d Dox withdrawal, pre-fear conditioning (**e**–**h**), and after recall (**i**–**l**). The rectangular area in **a**–**l** is magnified to demonstrate localization of ChR2-mCherry and c-Fos in representative neurons. **m** Number of DG granule neurons in sections near the injection site expressing endogenous c-Fos and ChR2-mCherry (one-way ANOVA, *n* = 4, 9, 16 per group). **n**. Co-expression of c-Fos and ChR2-mCherry after 2 d Dox withdrawal (Pre-Fear Conditioning), and after Recall (one-way ANOVA, *n* = 4, 9, 16 per group). Scale bar for fluorescent images: 200 µm

To confirm the inducible and activity-dependent expression of ChR2-mCherry in the contextual fear conditioning (CFC) paradigm, we examined its expression at different time points of testing. In mice that were on Dox while exposed to a novel context A, we observed a number of c-Fos positive DG neurons, but a complete absence of ChR2-mCherry expression (Habituation; Fig. 2a–d, m).

Two days of  Doxwithdrawal was sufficient to induce ChR2-mCherry expression in mice that were kept in their home cage (pre-fear conditioning; Fig. 2e–h, m). The number of ChR2-mCherry expressing neurons was increased significantly in mice after 2 days of Dox withdrawal followed by fear conditioning (Recall; Fig. 2i–l, m). We found that in mice that experienced light stimulation (Recall), the vast majority of ChR2-mCherry-expressing cells were also c-Fos-positive, indicating that stimulation of ChR2-mCherry activity through light stimulation was sufficient to induce neuronal activity (Fig. 2i–l, n).

To examine whether optical stimulation evoked responses from DG neurons expressing ChR2-mCherry or mCherry (control), we injected c-fos tTA mice with AAV_9_-TRE-ChR2-mCherry or AAV_9_-TRE-mCherry, trained them through fear conditioning (FC) while off Dox, then returned them to Dox diet for 24 h. A sparse subpopulation of DG neurons responded to optical stimulation (473 nm, 20 Hz, 15 ms) in the c-fos-tTA mice injected with AAV_9_-TRE-ChR2-mCherry, consistent with the sparse labeling of neurons in expression timeline experiments (Fig. 2i–l).

**Fig. 3:**
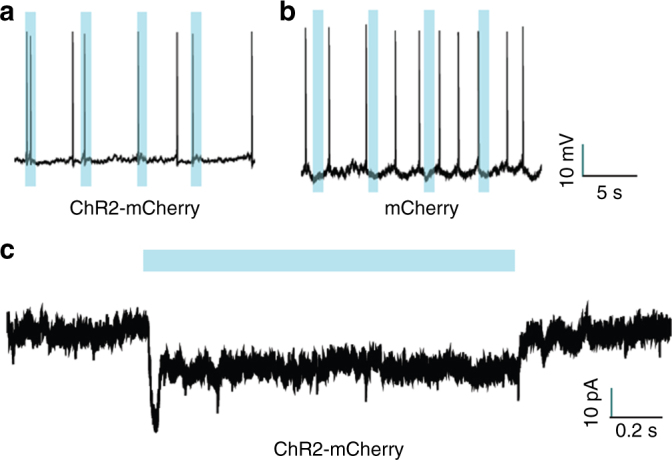
Stimulation of ChR2-mCherry induces neuronal activity. **a**, **b** Representative whole-cell current clamp recordings of neurons expressing ChR2-mCherry (**a**) or mCherry (**b**). **c** Representative whole-cell voltage clamp recording of neuron expressing ChR2-mCherry. Blue: 473 nm light stimulus

A reliable spike in response to the onset of light pulses was detected in ChR2-mCherry-expressing neurons (Fig. 3a). We did not detect any spike in response to optical stimulation in the c-fos-tTA mice injected with the control AAV_9_-TRE-mCherry (Fig. 3b). In addition, we detected a current of ~15 pA in response to optical stimulation in neurons expressing ChR2-mCherry (Fig. 3c).

### Behavioral validation of optogenetics model

Next, we examined whether optical stimulation of a subpopulation of DG neurons labeled with ChR2-mCherry during the acquisition of fear memory was sufficient for memory recall. c-fos-tTA mice were bilaterally injected with AAV_9_-TRE-ChR2-mCherry or AAV_9_-TRE-mCherry in the DG and implanted with an optical fiber immediately above the injection site (Supplementary Fig. 1a). Mice were treated with Dox while recovering from surgery, then underwent 1 day of Habituation in Context A to record basal freezing during exploration in both light-off and light-on periods. Dox was withdrawn for 24 h, then mice underwent fear conditioning in Context B in which mice were shocked towards the end of exploration. The mice were immediately placed back on 1 g kg^−1^ Dox for 24 h, then tested in context A under light-off and light-on periods (Supplementary Fig. 1b). During the Habituation session, mice in both groups displayed very little freezing during the light-off and light-on epochs (Supplementary Fig. 1c). During the Recall session after FC, mice injected with AAV_9_-TRE-ChR2-mCherry showed significantly higher freezing levels during light-on periods compared to light-off periods, indicating light stimulation-induced recall of fear memory (Supplementary Fig. 1c, Supplementary Fig. 1e). Mice injected with AAV_9_-TRE-mCherry showed no increase in freezing in the light-on period of the Habituation or Recall session (Supplementary Fig. 1d, e). Freezing was only induced in the Recall session for mice injected with AAV_9_-TRE-ChR2-mCherry (Supplementary Fig. 1e).

### Treatment with dietary polyphenols enhances recall of fear memory

**Fig. 4:**
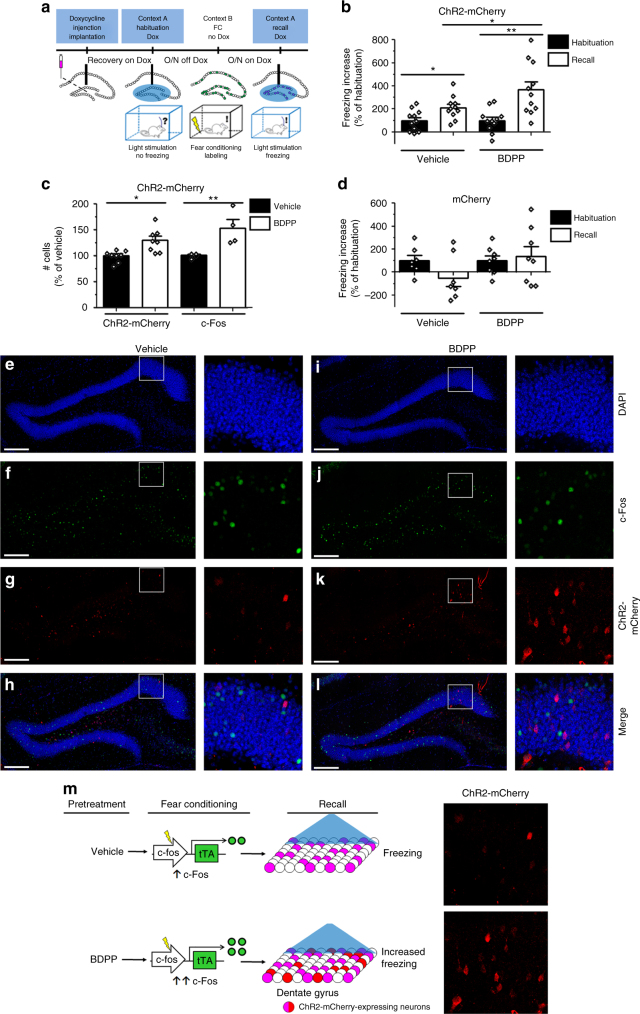
Treatment with BDPP promotes fear memory recall through upregulation of c-Fos. **a** Training scheme. After recovery from surgery, mice are treated with BDPP or vehicle for 2 w prior to training in CFC. **b** Increase in freezing upon light stimulation in Habituation and Recall sessions for mice injected with AAV_9_-TRE-ChR2-mCherry. Untreated and BDPP-treated mice both show an increase in freezing behavior in the Recall session, but the increase is greater in mice treated with BDPP. Recapitulation of freezing is normalized to the increase in freezing in the light-on period of the Habituation session (100%; one-way ANOVA, *n* = 11 per group). **c** Number of neurons in the DG of hippocampal sections from untreated and BDPP-treated mice expressing c-Fos and ChR2-mCherry (one-way ANOVA, *n* = 4,8 per group). **d** Increase in freezing upon light stimulation in Habituation and Recall sessions for mice injected with AAV_9_-TRE-mCherry. Mice do not show an increase in freezing behavior upon light stimulation in the Habituation or Recall session, regardless of treatment. Recapitulation of freezing is normalized to the increase in freezing in the light-on period of the Habituation session (100%; one-way ANOVA, *n* = 7–8 per group). **e**–**l** Representative images of the DG of hippocampal sections from vehicle-treated (**e**–**h**) and BDPP-treated (**i**–**l**) mice. The rectangular area in **e**–**l** is magnified to demonstrate localization of ChR2-mCherry and c-Fos in representative neurons. Scale bar: 200 µm. **m** Mechanism through which dietary polyphenols promote recruitment to a ChR2-mCherry-labeled memory engram. Treatment with dietary polyphenols stimulates *c-fos* promoter activity in the DG, resulting in expression of ChR2-mCherry upon withdrawal of Dox and training through CFC. Upon light stimulation in the Recall session, more neurons are labeled with ChR2-mCherry, resulting in increased freezing during the light-on period

To examine whether dietary polyphenols enhance optical recall of fear memory, we subjected a parallel group of c-fos-tTA mice injected with AAV_9_-TRE-ChR2-mCherry to treatment with BDPP or drinking water (vehicle) and tested them in the CFC paradigm (Fig. 4a). Both vehicle-treated and BDPP-treated mice showed very little freezing during the light-off and light-on periods of the Habituation session (Fig. 4b).

In the Recall session after FC, both vehicle-treated and BDPP-treated mice showed increased freezing in the light-on period; the increase of freezing was significantly higher in BDPP-treated mice, indicating promotion of light-induced fear memory recall (Fig. 4b). In a parallel group of c-fos-tTA mice injected with a silent AAV_9_-TRE-mCherry as a negative control and subjected to treatment with BDPP or vehicle, no increase of freezing was detected in the light-on period of the Habituation or the Recall session after FC (Fig. 4d), indicating that increased freezing in the mice expressing ChR2-mCherry was not due to non-specific effects of post-training optical stimulation.

In BDPP-treated mice, we detected an increased number of c-Fos- and ChR2-mCherry-expressing neurons compared to vehicle-treated mice (Fig. 4c, e–l). These data suggest that dietary BDPP treatment is able to deliver brain bioavailable polyphenolic metabolites (Supplementary Table 1, Supplementary Table 2) to enhance expression of endogenous c-Fos during training, resulting in increase of freezing upon optical stimulation as a result of labeling of a larger subpopulation of neurons with ChR2-mCherry (Fig. 4m).

## Discussion

We have shown through an optogenetics model that dietary polyphenols in the form of BDPP promote recruitment of DG granule neurons to fear memory through enhanced expression of c-Fos, as reflected by increased labeling of recently activated neuronal subpopulations with ChR2-mCherry (Fig. 4). Optogenetic stimulation of neurons influenced by BDPP recalls fear memory resulting in freezing behavior in the DG-dependent CFC paradigm, satisfying the requirement for sufficiency to demonstrate that this subpopulation of neurons forms a new component of a functional memory engram^30,31^. To our knowledge, this is the first demonstration of a causal relationship between the beneficial mechanisms modulated by dietary polyphenols in a select subpopulation of neurons and performance in hippocampus-dependent behavioral tasks.

Chemical analysis of BDPP reveals a complex mixture of phenolic compounds, including proanthocyanidins, flavan-3-ols, anthocyanidins, flavonols, and phenolic acids (Supplementary Table 1). Dietary polyphenols are extensively metabolized through xenobiotic and colonic microbiota-mediated metabolism, resulting in the generation of phenolic metabolites including polyphenolic conjugates and phenolic acids^5,32–34^. Analysis of plasma and brain tissue reveals that BDPP treatment delivers a number of phenolic metabolites in nanomolar concentration (Supplementary Table 2) which are previously shown to promote hippocampal CREB activity^7^. This suggests that BDPP treatment may deliver individual polyphenolic metabolites that are able to enhance c-Fos expression through activation of CREB and transcription of target genes, resulting in increased recruitment of granule neurons to memory^28^.

Lesion studies and optogenetic manipulations of hippocampal subregions demonstrate that the CA1, CA3, and DG are all required for the retrieval of spatial and fear memory, though there is no evidence, to our knowledge, that activation of the CA1 or CA3 is sufficient to recall fear memory and recapitulate freezing^14,16,35,36^.

Differentiation between similar environmental contexts is dependent on the sparse distribution of activated DG granule neurons^14,18^. Conditions of stress that negatively impact synaptic plasticity impair performance in the CFC paradigm, even when previously-activated DG granule neurons are activated through optogenetic stimulation^21,37^. As these deficits can be rescued through treatment with dietary polyphenols and activation of context-related DG neurons is sufficient to recapitulate behavior associated with memory, we focused on modulations of DG granule neurons by dietary polyphenols^10,12,19^. Other brain regions have been found to be critical for encoding and retrieval of contextual fear memories besides the hippocampus, including the basal and central amygdala and medial prefrontal cortex^38,39^. As treatments with dietary polyphenols mediate benefits in various functions in these regions, it would be intriguing to investigate whether the c-fos-tTA/TRE-ChR2 optogenetics model could also be adapted to these regions and map responses of neurons to dietary polyphenols^40,41^.

Our study focuses only on performance in the CFC paradigm. In our study, we noticed a general increase in freezing across all treatment groups (~15%) from the Habituation to Recall session (Supplementary Fig. 1). This suggests an increase in anxiety levels induced by fear conditioned that could not be eliminated by our experimental design and could not be examined through anxiety-related tests in our paradigm. The function of DG granule neurons plays a role in a number of behavioral tests, including spatial memory tests such as the Morris Water Maze and Conditioned Place Preference tests^42,43^. As optogenetic manipulations of the DG have been found to modulate behavior responses in the Conditioned Place Preference and Place Avoidance tasks, it would be intriguing to investigate whether dietary polyphenols are able to enhance recapitulation of behavior in tests with a spatial component^44^. Expanding the use of the c-fos-tTA/TRE-ChR2 optogenetics model to similar tests would eliminate confounding factors such as anxiety and map responses of subpopulations to dietary polyphenols in alternate behavioral tests.

Through optogenetic manipulations, we have demonstrated that certain dietary polyphenols are able to deliver bioactive metabolites to the brain to promote memory function through the stimulation of c-Fos expression in DG granule neurons, and influence neurons to form a novel domain of a functional memory engram. In summary, our findings suggest a general method in which the c-fos-tTA/TRE-ChR2 inducible optogenetics model can be used to study the mechanisms through which dietary polyphenols confer benefits in memory function and identify neuronal subpopulations in various brain regions influenced by treatment.

## Methods

### Subjects

c-fos-tTA mice were obtained from The Jackson Laboratory (#018306). Mice were given food and water ad libitum and were socially housed prior to surgery on a 12:12-h light/dark cycle with lights on at 07:00 h in a temperature-controlled (20 ± 2 °C) vivarium. Male mice were 8–14 weeks old at the time of surgery and had been raised on food containing 40 mg kg^−1^ Doxycycline (Custom Animal Diets) for 2 weeks before surgery. Mice were single-housed post-surgery. All procedures were approved by the Institutional Animal Care and Use Committee of the Icahn School of Medicine at Mount Sinai.

### Virus constructs

The pAAV_9_-TRE-ChR2-mCherry and pAAV_9_-TRE-mCherry plasmids were a kind gift from Dr. Susumu Tonegawa (Massachusetts Institute of Technology). These plasmids were used to generate AAV_9_ viruses by Virovek with assistance from the Viral Gene Transfer Core at the Massachusetts Institute of Technology. Viral titers used were 1.1 × 10^13^ vg mL^−1^ for AAV_9_-TRE-ChR2-mCherry and 1.0 × 10^13^ vg mL^−1^ for AAV_9_-TRE-mCherry.

### BDPP treatment

After injection with AAV_9_-TRE-ChR2-mCherry or AAV_9_-TRE-mCherry (control), c-fos-tTA mice were randomly grouped into two groups: one group received regular drinking water (vehicle), and the other was treated with BDPP composed of GSPE (MegaNatural®-BP Polyphenolics), RSV (ChromaDex), and CGJ (Welch Foods), delivered through drinking water for 2 weeks prior to experiments. The daily intake of GSPE was 200 mg kg^−1^ body weight (BW), RSV was 300 mg kg^−1^ BW, and CGJ was 1 mL d^−1^.

### Stereotactic injection and optical fiber implant

Implantations and injections were performed under stereotaxic guidance (Kopf). Briefly, mice were anesthetized with 100 mg kg^-1^ ketamine and 10 mg kg^−1^ xylazine. The virus was bilaterally injected with a 5 µL microsyringe using a 33-gauge needle (84851, Hamilton). After a bilateral craniotomy using a 0.7 mm burr (Fine Science Tools), the needles were slowly lowered to the target site (−2.0 mm anterioposterior (AP), ± 1.5 mm mediolateral (ML), −1.7 dorsoventral (DV)) and remained in place for 5 min prior to the beginning of the injection. Mice were injected with 0.5 µL of AAV_9_ virus at a rate of 0.1 µL min^−1^. The needles remained in place for 5 min before they were slowly withdrawn. Next, two jewelry screws (Plastics One) were screwed into the skull anterior and posterior to the injection site to provide additional anchor points for the implant. Fiber-optic cannulas (200 µm core diameter, Doric Neuro) cut to the optimal length (1.4 mm) were lowered directly above the injection site. Layers of dental cement (Teets Cold Cure, A-M Systems) were applied to secure the optical fiber implant. Mice remained on a heating implant until fully recovered from anesthesia and were given 1.5 mg kg^−1^ ketoprofen as analgesic for 48 h. Mice were singly-housed post-surgery and allowed to recover for 2 weeks prior to experiments. Sites of fiber implants and viral injects were verified histologically and we only included mice with ChR2-mCherry or mCherry expression isolated to the DG in our results.

### ChR2-mCherry expression timeline

Two weeks after surgery, mice underwent CFC protocols as described below. Mice were sacrificed at the following timepoints to establish a timeline of ChR2-mCherry expression: after the Habituation session; after 2 days of Dox withdrawal (pre-fear conditioning); 1 day after FC, immediately after the Recall session.

### Electrophysiology

Electrophysiological studies were performed 1 day after training in Fear Conditioning. Mice were anesthetized through isoflurane inhalation, decapitated, and brains were removed. Coronal slices (250 µm thick) were taken using a microslicer in a cutting solution oxygenated with 95% O_2_ and 5% CO_2_ (254 mM sucrose, 3 mM KCl, 1.25 mM NaH_2_PO_4_, 10 mM d-Glucose, 24 mM NaHCO_3_, 2 mM CaCl_2_ and 2 mM MgCl_2_; pH 7.35, 295–305 mOsm). Slices were incubated in a holding chamber filled with the cutting solution for 1 h at 37 °C. Slices were then transferred to a recording chamber with a constant flow rate of ACSF oxygenated with 95% O_2_ and 5% CO_2_ at 35 °C (flow rate = 2.5 mL min^−1^). Borosilicate glass microelectrodes consisting of patch pipettes (7–9 MΩ) filled with internal solution were used for whole-cell recordings (containing 115 mM potassium gluconate, 20 mM KCl, 1.5 mM MgCl_2_, 10 mM phosphocreatine, 10 mM HEPES, 2 mM magnesium-ATP and 0.5 mM GTP (pH 7.2, 285 mOsm). Slices were examined to confirm viral injection in the DG before recording. Recordings were amplified using a Multiclamp 700B Amplifier (Axon Instruments) and data acquisition was perfurmed using Digidata 1440 A and pClamp10 (Molecular Devices). Access resistance (Ra) was monitored throughout the recordings and data acquisition was suspended when resting membrane potential was depolarized above −50 mV or the Ra was above 20MΩ. Optogenetic stimulation was achieved using a 473 nm laser (30 mW; OEM Laser Systems) and a pulse train generator. In current-clamp mode, neurons were measured at resting potential and were stimulated by a sequence of twenty 15 ms pulses at 20 Hz every 5 s. In voltage-clamp mode, neurons were voltage-clamped at −70 mV and stimulated with blue light for 3 s.

### Immunohistochemistry

1.5 h after behavioral trials, mice were anesthetized with ketamine/xylazine and perfused transcardially with cold PBS, followed with 4% paraformaldehyde (PFA) in PBS. Brains were removed and kept in 4% PFA at 4 °C overnight. Coronal slices (40 µm thick) were taken using a vibrotome and collected in cold PBS + 0.02% sodium azide. For immunostaining, each slice was washed three times with cold PBST (PBS + 0.1% Triton X-100) for 10 min, then placed in PBST with 5% normal goat serum for 1 h. Slices were then incubated with primary antibody at 4 °C for 24 h (Rabbit anti-RFP (600-401-379) 1:500, Rockland; Chicken anti-GFP (A10262) 1:500, Invitrogen; Mouse anti-c-Fos (sc-166940) 1:200, Santa Cruz). The next day, slices were washed three times for 10 min in cold PBST, followed by 1 h incubation in secondary antibody (Goat anti-Mouse AlexaFluor647 (115-605-146) 1:200, Jackson ImmunoResearch; Goat anti-Chicken AlexaFluor488 (103-545-155) 1:200, Jackson; Goat anti-Rabbit AlexaFluor568 (ab175471), 1:200, Abcam). Sections were washed three more times for 10 min in cold PBST, followed by mounting and coverslipping on microscope slides using VectaShield containing DAPI (H-1500, Vector Laboratories). Images of coronal sections were taken on a Zeiss LSM880 Airyscan under an X20/0.8 NA air immersion objective controlled by Zeiss Zen Black software. Microscopy and/or image analysis was performed at the Microscopy CoRE at the Icahn School of Medicine at Mount Sinai.

### Behavioral assays

All behavioral assays were conducted 1 h after the beginning of the light cycle of the day. Two contexts were used in the behavioral assays. Context A was a 30.5 × 24.1 × 21 cm conditioning chamber (Med Associates) within a room with white walls and dim lighting. The chamber had a white plastic floor, vertical striped wallpaper, dim lighting, low background fan noise, and was scented with 0.25% benzaldehyde. Context B was a 30.5 × 24.1 × 21 cm conditioning chamber within the same room with bright lighting. The chamber had a metal rod floor, grey walls, high background fan noise, bright lighting, and was scented with 1% acetic acid. All experimental groups underwent training under the same protocol. For 3 days, mice were acclimated to handling and having implants connected to fiber optic patch cords in an anteroom. During the Habituation session, mice were exposed to context A for 1 day while on 40 mg kg^−1^ Dox. The fiber optic implant was connected to a 460 nm fiber-coupled LED (Prizmatix) controlled by a pulse train generator (Prizmatix). The mice were allowed to freely explore context A for 4 min. The Habituation session was divided into a 2-min light-off period, followed by a 2-min light-on period. The mouse received optical stimulation (9 mW, 20 Hz, 15 ms) throughout the entire period. At the end of the session, the mouse’s implants were disconnected from the patch cords and the mouse was returned to its home cage. After the Habituation session, the mouse was kept on regular food without Dox for 24 h until the Fear Conditioning session. During the Fear Conditioning session, the mouse was introduced to context B for 360 s. The mouse received a footshock (2 s, 0.75 mA) at 180, 240, and 300 s. After the trial, the mouse was returned to its home cage and placed on food containing 1 g kg^−1^ Dox overnight. The Recall session started the next day and was conducted the same as the Habituation session in context A. Mice were sacrificed 1.5 h after testing for perfusion and immunohistological analysis. Freezing behavior in all sessions was recorded with a side-mounted NIR camera and measured with Any-Maze software (Stoelting).

### Statistical analysis

All values are presented as mean and standard error of the mean (s.e.m.). For cell counting, one-way ANOVA was used to compare all groups followed by Bonferroni’s comparison of testing group and the control group (confidence interval = 95%). For behavioral assays, one-way ANOVA followed by Bonferroni’s comparison or unpaired two-tailed student’s *t*-test with Welch’s correction was used (confidence interval = 95%). In all studies, outliers (2 SD from the mean) were excluded. All statistical analysis was performed using GraphPad Prism 5 software (GraphPad Software). *p < 0.05, **p < 0.01, ***p < 0.001, n.s. not significant.

### Data availability

The data that support the findings of this study are available from the corresponding author upon reasonable request.

## Electronic supplementary material

Supplementary Information
