# Supplementary material for: Dietary polyphenols enhance optogenetic recall of fear memory in hippocampal dentate gyrus granule neuron subpopulations
Source: Commun Biol. 2018 May 3;1:42. doi: 10.1038/s42003-018-0043-5 (PMC6123622; doi:10.1038/s42003-018-0043-5)
Supplement: Supplementary file 1 — Supplementary Information [file 42003_2018_43_MOESM1_ESM.pdf]

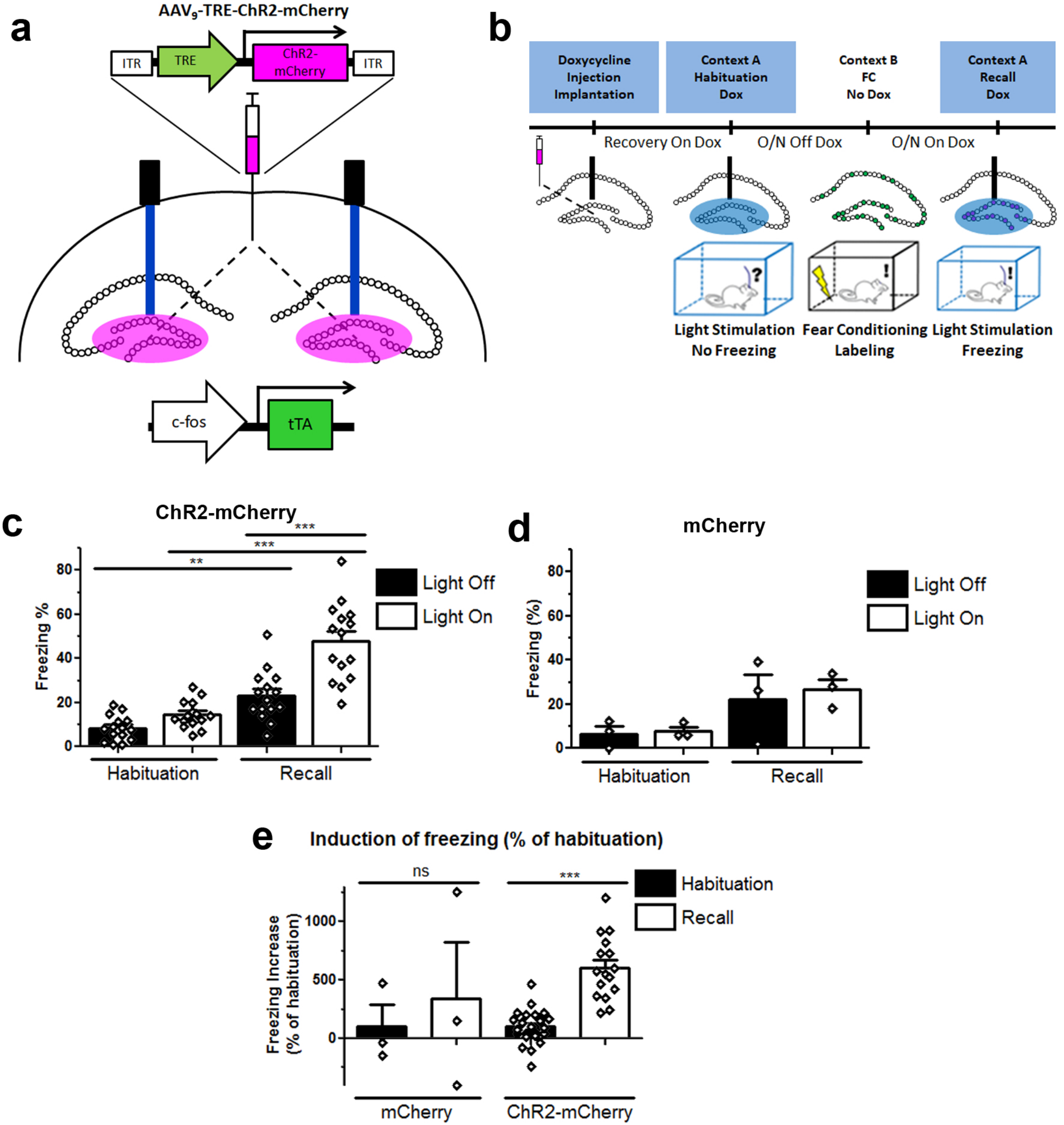
a
AAV9-TRE-ChR2-mCherry
ITR
TRE
ChR2-mCherry
ITR
c-fos
tTA
b
Doxycycline Injection Implantation
Context A Habituation Dox
Context B FC No Dox
Context A Recall Dox
Recovery On Dox
O/N Off Dox
O/N On Dox
Light Stimulation No Freezing
Fear Conditioning Labeling
Light Stimulation Freezing
c
ChR2-mCherry
Freezing %
Light Off
Light On
Habituation
Recall
d
mCherry
Freezing (%)
Light Off
Light On
Habituation
Recall
e
Induction of freezing (% of habituation)
ns
***
Habituation
Recall
Freezing Increase (% of habituation)
mCherry
ChR2-mCherry

Supplementary Figure 1: Light stimulation of ChR2-mCherry-expressing neurons recapitulates freezing behavior in the CFC paradigm. **a.** c-fos-tTA mice are bilaterally injected with AAV_9-TRE-ChR2-mCherry or AAV_9-TRE-mCherry, then implanted with fiber optic cannulas directly above the injection site. **b.** Scheme of CFC paradigm. After recovery from surgery, mice are habituated to Context A during light-off and light-on epochs (Habituation). Dox is withdrawn for 1 d and mice are trained via footshock in Context B (FC). Mice are returned to Dox and tested in Context A for light-induced freezing (Recall). **c-e.** Recapitulation of freezing upon light stimulation in the Habituation and Recall session. Freezing behavior in mice expressing ChR2-mCherry (**c**) or mCherry (**d**). **e.** Increase of freezing during the light-on epoch, normalized to the increase during the Habituation session (one-way ANOVA, n = 3,15 per group).

a

BDPP Component	Phenolic compound class<br>Phenolic compound name	Concentration (mg/gm CGJ polyphenol)*
**Concord Grape Juice (CGJ)**		
	**Proanthocyanidins (PA)**	
	PA-dimers	59.8
	**Flavan-3-ols**	
	Catechin	45.2
	Epicatechin	26
	**Anthocyanidins (Acs)**	
	Cyanidin-G **	56.9
	Cyanidin-G-Ac **	5.4
	Cyanidin-G-Co **	9.5
	Cyanidin-G-G-Co **	27.1
	Delphinidin-G **	74.4
	Delphinidin-G-Ac **	45.6
	Delphinidin-G-Co **	21.7
	Malvidin-G **	16.3
	Malvidin-G-Co **	3.6
	Malvidin-G-G **	76.3
	Malvidin-G-G-Co **	28.9
	Peonidin-G-Co **	1.2
	Peonidin-G--G-Co **	13.4
	Petunidin-G-Ac **	16.1
	Putinidin-G-Co **	6
	**Flavonols**	
	Malvidin-G **	4.9
	Quercetin-G **	3.9
	Quercetin-Gln **	4.2
	Quercetin-3-O-Rutinoside	5.9
	**Phenolic Acids**	
	Gallic Acid	4.2

* Unpublished observation

** G, glucosyl or galactosyl moiety; Ac, acetyl; Co, coumaroyl

b

BDPP Component	Phenolic compound class<br>Phenolic compound name	Concentration (mg/gm GSPE polyphenol)*
**Grape Seed Polyphenol Extract (GSPE)**		
	**Proanthocyanidins (PA)**	
	PA-dimers	79.2
	**Flavan-3-ols**	
	Catechin	33.7
	Epicatechin	17
	**Phenolic acids**	
	Gallic acid	22.3

* Unpublished observation

c

BDPP Component	Phenolic compound class<br>Phenolic compound name	Concentration (mg/gm RSV polyphenol)*
**Resveratrol (RSV)**		
	**Stilbenoids**	
	Resveratrol	999.9

* Unpublished observation

Supplementary Table 1: Polyphenol contents of BDPP components. BDPP is comprised of three commercially available components, a select Concord Grape Juice (CGJ), a select Grape Seed Polyphenol Extract (GSPE), and all trans-resveratrol (RSV). In a series of quality control studies, polyphenol contents of CGJ, GSPE, and RSV were analyzed by LC/UV-MS/MS[1]. **a-c**. Names and concentrations of polyphenols and phenolic acids identified from CGJ (**a**), GSPE (**b**), and RSV (**c**). Phenolic compounds are clustered by their polyphenol structural classes.

**Bioavailability of phenolic metabolites from a Bioactive Dietary Polyphenol Preparation (BDPP; 783 mg polyphenol / kg BW / day) in plasma and perfused brain specimens from rats #**		
**Polyphenol metabolites**	**Plasma Concentration (μM)**	**Brain Concentration (pmol/g)**
**Flavan-3-ols**		
catechin-5-O-glucuronide	2.46 ± 0.26	485.79 ± 85.07
epicatechin-5-O-glucuronide	2.79 ± 0.19	637.22 ± 93.85
3'0Me-Catechin-5-0-Glucuronide	2.82 ± 0.07	664.29 ± 133.65
3'0Me-Epicatechin-5-0-glucuronide	4.2 ± 0.13	853.83 ± 142.77
**Anthocyanidins**		
cyanidin-glucoside	0.01 ± 0.001	0.07 ± 0.00
delphinidin-glucoside	0.004 ± 0.0003	0.07 ± 0.00
malvidin-glucoside	0.004 ± 0.0004	0.17 ± 0.02
peonidin-glucoside	0.004 ± 0.0002	0.12 ± 0.01
petunidin-glucoside	0.003 ± 0.0005	0.10 ± 0.00
**Flavonols**		
quercetin-3-O-glucuronide	0.11 ± 0.04	2.41 ± 0.47
Ome-quercetin-O-glucuronide	0.079 ± 0.008	0.69 ± 0.05
**Stilbenoids**		
resveratrol	NA *	NA *
resveratrol-3-O-glucuronide	78.53 ± 3.24	746.57 ± 121.73

Adapted from Wang et al., Front Aging Neurosci. 6:42, 2014

* NA, not assessed

Supplementary Table 2: Biologically available phenolic metabolites from BDPP. Rats were treated orally with BDPP. On the last day, mice were rats the final dose by gavage followed by collection of plasma. Plasma content of phenolic compounds was analyzed by LC-MS/MS[2,3]. Concentration of polyphenolic compounds from BDPP. Phenolic compounds are clustered according to their polyphenol structural classes. For each compound, we present values for plasma concentration and brain concentration. Values are mean ± SD.
